# 83649 Modeling COVID-19 infection dynamics and program interventions for K-12 school re-opening

**DOI:** 10.1017/cts.2021.469

**Published:** 2021-03-30

**Authors:** Douglas E. Morrison, Roch Nianogo, Vladimir G. Manuel, Onyebuchi A. Arah, Nathaniel Anderson, Tony Kuo, Moira Inkelas

**Affiliations:** 1 Fielding School of Public Health, University of California Los Angeles; 2 David Geffen School of Medicine, University of California Los Angeles

## Abstract

ABSTRACT IMPACT: This study provides public health and K-12 school districts with a pragmatic, flexible, adaptable model showing COVID-19 transmission dynamics, using local data and program elements that are modifiable and with an online model for easy use, to enable safe and equitable re-opening and maintenance of in-person learning. OBJECTIVES/GOALS: School closures resulting from the COVID-19 pandemic disrupt student education and health and exacerbate inequities. Public health agencies and school districts currently lack pragmatic models to assess the effects of potential strategies for resuming and maintaining in-person learning on outcomes such as transmission and attendance. METHODS/STUDY POPULATION: This study explored how various combinations of transmission-mitigating interventions affect health and learning outcomes in a range of underlying epidemiological conditions. The CTSA science team developed a conceptual framework and an agent-based simulation model with parameters including prevalence, transmission, testing, preventive and responsive actions, infection control, population behavior and awareness, and the potential impact of vaccine adoption and exemption policies. The team partnered with a large school district to ensure relevance of the program components to decision-making. RESULTS/ANTICIPATED RESULTS: The model shows that no single program element or condition ensures safety. Combining interventions can result in synergy in the mitigation efforts. Even without testing, an efficient health screening process with forthcoming risk reporting, combined with on-campus infection control, can reduce on-campus transmission. The resulting model is accessible online to enable exploration of likely scenarios. It is adaptable as COVID-19 science evolves, including for testing and vaccines. DISCUSSION/SIGNIFICANCE OF FINDINGS: This research provides public health agencies and school districts with a model that couples local conditions with programmatic elements to help inform the local COVID-19 response, recognizing that decisions about the school community are often complex politically, technically, and operationally when it comes to addressing a health crisis.

